# Pecans and Its Polyphenols Prevent Obesity, Hepatic Steatosis and Diabetes by Reducing Dysbiosis, Inflammation, and Increasing Energy Expenditure in Mice Fed a High-Fat Diet

**DOI:** 10.3390/nu15112591

**Published:** 2023-05-31

**Authors:** Claudia Delgadillo-Puga, Ivan Torre-Villalvazo, Lilia G. Noriega, Leonardo A. Rodríguez-López, Gabriela Alemán, Erik A. Torre-Anaya, Yonatan Y. Cariño-Cervantes, Berenice Palacios-Gonzalez, Janette Furuzawa-Carballeda, Armando R. Tovar, Luis Cisneros-Zevallos

**Affiliations:** 1 Departamento de Nutrición Animal Dr. Fernando Pérez-Gil Romo, Instituto Nacional de Ciencias Médicas y Nutrición Salvador Zubirán (INCMNSZ), Mexico City 14080, Mexico; 2 Departamento de Fisiología de la Nutrición, Instituto Nacional de Ciencias Médicas y Nutrición Salvador Zubirán (INCMNSZ), Mexico City 14080, Mexico; 3 Unidad de Vinculación Científica Facultad de Medicina, Instituto Nacional de Medicina Genómica (INMEGEN), Mexico City 16080, Mexico; 4 Departamento de Cirugía Experimental, Instituto Nacional de Ciencias Médicas y Nutrición Salvador Zubirán (INCMNSZ), Mexico City 14080, Mexico; 5 Department of Horticultural Sciences, Texas A&M University, College Station, TX 77843-2133, USA

**Keywords:** pecans, polyphenols, obesity, dysbiosis, insulin resistance, mitochondrial activity, functional food, bioactive compounds

## Abstract

Pecans (*Carya illinoinensis*) are considered a functional food due to the high content of polyunsaturated fatty acids, dietary fiber and polyphenols. To determine the effect of whole pecans (WP) or a pecan polyphenol (PP) extract on the development of metabolic abnormalities in mice fed a high-fat (HF) diet, we fed C57BL/6 mice with a Control diet (7% fat), HF diet (23% fat), HF containing 30% WP or an HF diet supplemented with 3.6 or 6 mg/g of PP for 18 weeks. Supplementation of an HF diet with WP or PP reduced fat mass, serum cholesterol, insulin and HOMA-IR by 44, 40, 74 and 91%, respectively, compared to the HF diet. They also enhanced glucose tolerance by 37%, prevented pancreatic islet hypertrophy, and increased oxygen consumption by 27% compared to the HF diet. These beneficial effects were associated with increased thermogenic activity in brown adipose tissue, mitochondrial activity and AMPK activation in skeletal muscle, reduced hypertrophy and macrophage infiltration of subcutaneous and visceral adipocytes, reduced hepatic lipid content and enhanced metabolic signaling. Moreover, the microbial diversity of mice fed WP or PP was higher than those fed HF, and associated with lower circulating lipopolysaccharides (~83–95%). Additionally, a 4-week intervention study with the HF 6PP diet reduced the metabolic abnormalities of obese mice. The present study demonstrates that WP or a PP extract prevented obesity, liver steatosis and diabetes by reducing dysbiosis, inflammation, and increasing mitochondrial content and energy expenditure. Pecan polyphenols were mainly condensed tannin and ellagic acid derivatives including ellagitannins as determined by LC-MS. Herein we also propose a model for the progression of the HF diet-mediated metabolic disorder based on early and late events, and the possible molecular targets of WP and PP extract in preventive and intervention strategies. The body surface area normalization equation gave a conversion equivalent to a daily human intake dose of 2101–3502 mg phenolics that can be obtained from 110–183 g pecan kernels/day (22–38 whole pecans) or 21.6–36 g defatted pecan flour/day for an average person of 60 kg. This work lays the groundwork for future clinical studies.

## 1. Introduction

Obesity is the major risk factor for metabolic syndrome, characterized by glucose intolerance, hepatic fat accumulation and increased circulating lipids and which if left untreated leads to chronic diseases such as cardiovascular diseases and type 2 diabetes mellitus. Several underlying causes of the metabolic derangements during obesity have been proposed, including adipose tissue dysfunction, increased hepatic lipogenesis, reduced skeletal muscle mitochondrial content and activity and microbiota dysbiosis (altered microbial diversity and composition). However, the temporal appearance and the prominence of each of these metabolic alterations is still not clear.

The US and Mexico are two of the countries with the highest rates of obesity worldwide. In average obesity prevalence among US adults was ~42.4% in the period 2017–2018 in both men and women [1] and overall estimated projections of ~50% adults by 2030 [2]. Similarly, in Mexico it was reported that >30% of the adult population was obese by 2018 with estimated projections of ~54% and 37% for men and women by 2050 [3]. Obesity represented an estimated cost and impact in the Mexican health care system of ~USD810 million in 2010 with an estimated projection of ~USD1.7 billion by 2050 [3]. In the US, obesity has a yearly cost of ~USD147 billion that represents 9% of the annual medical expenditure. Notably a 1 point % reduction of these projections for the following 20 years could represent medical expenditure reductions by ~USD84.9 billion [4,5]. The above numbers, and the fact that in both countries cardiovascular disease and type 2 diabetes are the leading cause of mortality, demand an intensification of efforts in studying the mechanism that leads from obesity to metabolic syndrome in order to develop novel preventive and therapeutic strategies to prevent these diseases. One of these strategies comprises the consumption of functional foods containing beneficial bioactive compounds such as fruits, vegetables, grains and nuts as part of the regular diet [6,7,8] that could potentially exert health-promoting effects against the metabolic syndrome [9].

Dietary patterns are one of the major contributors to the development of obesity. The Western diet has been associated with an increased risk of obesity and related health problems. This dietary pattern is characterized by a high intake of energy-dense foods, including processed meats, refined grains, sweets, and sugary drinks. These foods are typically low in fiber, vitamins, and polyphenols, but high in calories, saturated fats, and added sugars. On the other hand, healthy dietary patterns, such as the Mediterranean diet, have been associated with a reduced risk of obesity and related chronic diseases. The Mediterranean dietary pattern is characterized by a high intake of fruits, vegetables, whole grains, legumes, nuts, seeds, and healthy fats, such as olive oil and fish. This type of diet is rich in fiber, vitamins, and polyphenols, and low in saturated and trans fats and added sugars. The beneficial effects of a Mediterranean diet are mediated by antioxidant, prebiotic, immunomodulatory and nutrigenomic activities. In recent years, there has been a shift towards healthier dietary patterns in many countries, including the United States. This shift has been driven by a growing awareness of the health consequences of a poor diet and by public health campaigns promoting healthy eating.

Pecan nuts originally from the US and Mexico have also received much attention due to their unique chemistry of condensed and hydrolysable tannins, polyunsaturated fatty acids and high antioxidant levels [10,11], as well as to clinical studies showing a decrease in LDL oxidation [12], LDL cholesterol lowering effects [13], and improvement of cardiometabolic risk factors [14]. However, these studies were based on a limited dose range of ~42–90 g pecan/day, which corresponds to the FDA recommended daily consumption of nuts of 42.5 g [15] or approximately twice this amount.

Daily polyphenolic intake in humans ranges from 183–4854 mg/day associated with the consumption of a range of food items including coffee, tea, vegetables and fruit among others [16]. Interestingly, according to Villarreal-Lozoya et al. [11], different pecan varieties on average have a phenolic content of ~1520 mg/100 g pecans (determined from 76 mg chlorogenic acid equivalent/g defatted pecans and 80% oil content). Thus, the 42–90 g pecan intake previously reported in clinical studies would correspond to a daily dose of ~638–1368 mg of phenolic compounds. Accordingly, we hypothesize that pecan biological properties have been underestimated in previous clinical studies due to the low intake of pecan-derived bioactive phenolics.

In the present study we defined a framework for the progression of metabolic disorders during diet-induced obesity as early and late events to assess the biochemical, histological and molecular effects of whole pecans and pecan phenolic extracts on the development of early and late alterations in metabolic organs of mice through a preventive strategy and an interventionary one.

## 2. Materials and Methods

### 2.1. Whole Pecans, Pecan Phenolic Extract Preparation and Phenolic Quantification

Whole pecans of the “Pawnee” variety were kindly provided by Royalty Pecan Farms (Caldwell, TX, USA). The weight range of the pecan kernels was ~1.43 to 2.58 g (1 half kernel). To obtain pecan phenolic extracts, batches of ~580 g pecan kernels were blended in a mixer to obtain a sticky paste. The pecan paste was defatted with 6 L of hexane (2 times extraction) with stirring, and afterwards the hexane was separated using a Buchner funnel with filter paper (size P15), and the defatted cake (114.3 g) was allowed a 15 h evaporation of the remaining hexane to obtain a defatted pecan flour. The oil content was ~80.37% of the kernels. The defatted pecan flour was extracted with 3 L of aqueous acetone (70% acetone) for 15–17 h with stirring. The obtained extract was separated using a Buchner funnel and filter paper (size P15) and partially evaporated in a rotavapor (40 °C at 22–24 Hg mm under vacuum), and afterwards it was dried using a freeze drier to yield ~25.7 g of a rich pecan phenolic extract. The phenolic content was assayed using the Folin-Ciocalteu phenol reagent as described by Villarreal-Lozoya et al. [11]. The phenolic concentration of the whole pecan kernels was ~1908.03 mg/100 g kernels (or ~9719.96 mg/100 g defatted pecan flour), while the phenolic content in the pecan phenolic extracts ranged from ~378–455 mg/g extract. The phenolic content was expressed as mg of chlorogenic acid equivalents determined by a standard curve. Antioxidant activity (AOX) was determined by the Oxygen Radical Absorbance Capacity (ORAC) assay as described by Villarreal–Lozoya et al. [11] indicating that whole pecans had an AOX of ~301.63 μmol Trolox equivalents/g kernels. The sample extracts were re-dissolved in methanol for the Liquid chromatography–mass spectrometry (LC-MS) analysis, 10 μL was injected with a concentration of 10 mg/mL.

### 2.2. LC-MS Phenolic Profiling

The determination of individual compounds was performed on a Surveyor HPLC/MS system equipped with an autosampler, a Surveyor 2000 quaternary pump, and a Surveyor UV 2000 PDA detector using a C18 reverse phase (150 mm × 4.6 mm, Atlantis, Waters, Ireland; particle size = 5 μm) column connected to a LCQ Deca XP Max MS^n^ system (Thermo Finnigan, San Jose, CA, USA) with a Z-spray ESI source run by Xcalibur software, version 1.3 (Thermo Finnigan-Surveyor, San Jose, CA, USA). The mobile phase flow rate was set at 0.25 mL/min, while the elution gradients were performed with solvent A, consisting of acetonitrile/methanol (1:1) (containing 0.5% formic acid); and solvent B, consisting of water (containing 0.5% formic acid). The applied elution conditions were: 0–2 min, 2% A, 98% B; 3–5 min, 5% A, 95% B; 5–7 min, 25% A, 75% B; 7–12 min, 55% A, 45% B; 12–24 min, 55% A–80% A, 24–27 min held isocratic at 80% A, 28–30 min 90% A, 10% B; 31–33 min held isocratic, 100% A; 34–40 min, 2% A, 98% B, to the starting condition. The chromatograms were monitored at 280 nm, and complete spectral data were recorded in the range 200–600 nm. ESI was performed in the negative ionization mode, nitrogen was used as sheath gas with a flow of 59 arbitrary units, and He gas was used as dampening gas. The capillary voltage, −4.17 V; spray voltage, 5 kV; capillary temperature, 275 °C; and tube lens voltage at −55 V. Collision energies of 30% were used for the MSn analysis.

### 2.3. Experimental Diets

All diets were prepared using purified ingredients according to the guidelines of the American Institute of Nutrition (AIN-93) [17] (Table 1), where (1) the control diet (C) contained 7% *w*/*w* fat from soy oil, (2) the high-fat diet (HFD) contained 23% *w*/*w* fat from soy oil and lard, (3) the high-fat diet supplemented with whole pecan nuts (HF WP) contained 23% *w*/*w* fat from soy oil and pecan fat. The quantity of polyphenols in the HF WP diet was 5.7 mg phenolics/g diet. The high-fat diets with a pecan phenolic extract added contained 23% *w*/*w* fat from soy oil and lard, where (4) one diet was supplemented with 3.6 mg phenolics/g of diet (HF 3PP) and (5) another with 6 mg phenolics/g diet (HF 6PP). The ingredients were thoroughly mixed and pelletized. Experimental diets were prepared weekly. Polyphenols were extracted and quantified from Whole Pecans as previously described by Villarreal-Lozoya et al. [11]. The appropriate weight of pecan phenolic extract was calculated according to amounts of doses of polyphenol of each batch extracted. The composition of each diet is presented in Table 1.

### 2.4. Animal Study Design

In total, 42 male C57BL/6 mice were employed in the study and divided into 2 protocols, a preventive and a therapeutic (intervention) protocol. In the prevention protocol, 30 animals (3 weeks old and 19–22 g body weight) were randomly assigned to 5 treatments (*n* = 6); as follows, (1) control diet (C) with 7% of fat, (2) high-fat diet (HF) with 23% of fat, (3) high-fat diet with 23% fat from whole pecan nuts (HF WP), (4) high-fat diet supplemented with 3.6 mg/g of pecan polyphenols (HF 3PP) and (5) high-fat diet supplemented with 6 mg/g of pecan polyphenols (HF 6PP). In the therapeutic (intervention) protocol, 12 animals were randomly assigned to 2 treatments groups (*n* = 6) as follows: (1) high-fat diet (HF) with 23% fat, fed with HF diet for 22 weeks; and (2) HF feeding for 18 weeks and switched to the HF 6PP during the last 4 weeks.

The mice were housed under 12 h light/dark cycles with *ad libitum* access to water and their respective experimental diet for eighteen weeks. Food consumption was recorded five times a week and weight gain was registered once a week until the end of the study. At the 18th week, the mice were food-deprived for 6 h and euthanized with a sevoflurane overdose (Fluoromethyl-2,2,2-trifluoro-1-(trifluoromethyl) ethyl ether). Blood was collected through portal venipuncture in heparinized tubes and centrifuged at 3500 rpm, for 15 min at 4 °C. Plasma was collected and stored at −70 °C. The liver, pancreas, skeletal muscle (gastrocnemius and soleus) and subcutaneous, visceral and brown adipose tissues (SAT, VAT and BAT, respectively), were rapidly excised and divided into 2 fractions. One fraction was snap-frozen in liquid nitrogen and stored at −70 °C and the other fraction was fixed in ice-cold 4% (*w*/*v*) paraformaldehyde in phosphate buffer saline (PBS) for histopathological analysis. All animal procedures were conducted in accordance with the recommendations and procedures from the National Institutes of Health guide for care and use of Laboratory Animals [18]. The Animal Care Committee of the Instituto Nacional de Ciencias Médicas y Nutrición Salvador Zubirán (CICUAL-INCMNSZ) approved the study (Approval number NAN-1924-18-19-1).

### 2.5. Biochemical Parameters

Serum was immediately processed to determine fasting glucose, triglycerides and total cholesterol using a Beckman Coulter DxC 600 analyzer. Insulin, TNF-α and LPS concentrations in the serum were performed by ELISA (ALPCO, Salem, NH, USA, Cloud-Clone Corp, Katy, TX, USA, respectively) assays which were executed according to the manual provided with each kit. Serum samples by LPS were diluted using the provided standard diluent at a final proportion of 1:4 (*v*/*v*). The standard curve was plotted as follows: absorbance as a dependent variable and the logarithm of the concentration as an independent variable. A competitive inhibition model of analysis was selected to obtain curve equations and to interpolate the data. Results were corrected by dilution factor using GraphPad Prism 7.0 (GraphPad Software, San Diego, CA, USA).

### 2.6. Energy Expenditure and Body Composition Measurements

Energy expenditure was evaluated by indirect calorimetry in an Oxymax Lab Animal Monitoring System (CLAMS; Columbus Instruments, Columbus, OH, USA). The animals were individually housed in Plexiglas cages with an open flow system and continuously monitored for 24 h. The mice were food-deprived in the light period (12 h) and fed during the dark period (12 h). Oxygen consumption (VO_2_, mL/kg/h or mL/kg lean mass/h) and CO_2_ production (VCO_2_, mL/kg/h) were measured sequentially during 90 s throughout the 24 h test period. The respiratory exchange ratio (RER) was calculated as the average ratio of produced CO_2_ to O_2_ inhaled (VCO_2_/VO_2_). The data from the first 8 h were not considered for analysis as was the period for acclimatization to the cages. Additionally, a fecal sample (100–200 mg) of each animal was collected while housed, and frozen until analysis. Lean and fat mass content were determined in each mouse trough magnetic resonance (EchoMRI; Echo Medical Systems, Houston, TX, USA).

### 2.7. Intraperitoneal Insulin and Glucose Tolerance Test

The intraperitoneal insulin tolerance test (ipITT) was executed by the intraperitoneal administration of an insulin dose (0.5 UI/kg body weight). The intraperitoneal glucose tolerance test (ipGTT) was carried out by the intraperitoneal injection of a glucose dose (2 g/kg body weight). Both tests were performed in 6 h food-deprived mice. Blood glucose was assayed in a drop blood sample from the tail vein at 0, 20, 40, 60, 90, and 120 min after the glucose or insulin administration with a blood glucose monitoring system (Freestyle Optium, Abbott Laboratories, Lake Forest, IL, USA).

### 2.8. Histopathologic Evaluation of Hepatic, Pancreatic and Adipose Tissues

Formalin-fixed tissues were dehydrated in ethanol, cleared in xylenes and embedded in paraffin. Four µm sections were stained with hematoxylin and eosin (H&E) and analyzed using a Leica DM750 microscope (Leica, Wetzlar, Germany) at 20× and 40×. Adipocyte areas were identified and counted using Adiposoft software as previously described [19]. Pancreatic islet areas were evaluated using ImageJ software (National Institutes of Health, Bethesda, MD, USA). The software was calibrated with a 100 µm scale bar and the perimeter of each islet was manually drawn from two different images of each tissue.

### 2.9. Lipid Content in Liver and Skeletal Muscle

An analysis of lipid content in liver, soleus and gastrocnemius skeletal muscles was performed in 12 μm sections of snap-frozen samples embedded in optimal cutting temperature (OCT) compound and mounted in positively charged slides (Kling-On SFH1103, BIOCARE medical). Lipid accumulation in muscle fibers was observed by fixing frozen sections in ice-cold PBS-buffered 4% paraformaldehyde for 10 min, washing in deionized water and incubating with the lipophilic dye BODIPY 493/503 (790389, Sigma-Aldrich) (20 µg/mL in PBS) for 30 min. Slides were then washed in PBS, mounted using an aqueous mounting medium with DAPI (ProLong Gold Antifade Mountant P36931, Invitrogen) and observed under fluorescence illumination. Images of each section were taken at 20× magnification. The green BODIPY stain (lipids) and the blue DAPI stain (nuclei) were merged and quantified using ImageJ software (version 1.53t 24 August 2022).

### 2.10. Mitochondrial Activity in Skeletal Muscle

Analysis of mitochondrial activity in soleus and gastrocnemius skeletal muscles was also performed in 12 μm sections of snap-frozen samples embedded in optimal cutting temperature (OCT) compound and mounted in positively charged slides (Kling-On SFH1103, BIOCARE medical). Mitochondrial content was evaluated by succinate dehydrogenase (SDH)-nitro-blue tetrazolium (NBT) staining as described [20]. Frozen sections were incubated in SDH-NBT solution (0.05 mM sodium succinate, 0.55 mM nitro-blue tetrazolium) for 60 min at 37 °C. Slides were then washed in deionized water and dehydrated (2 min) by immersion in 30%, 60% and 90% acetone sequentially. Slides were rehydrated by immersion in 60% and 30% acetone in deionized water (2 min). Each section was photographed at 20× by bright-field microscopy. Mitochondrial abundance (blue stain) was quantified using Image J software as described [21].

### 2.11. Brown and White Adipose Tissues UCP-1 Content and F12/80 Abundance in Subcutaneous Adipose Tissue Macrophages by Immunohistochemistry

Immunostaining of BAT and SAT were performed in formalin-fixed, paraffin embedded tissues sectioned at 4 μm thick, according to Delgadillo-Puga et al. [22] with some modifications. Rehydrated slides were incubated with 3% H_2_O_2_ solution to block endogenous peroxidase activity, followed by incubation with IHC background blocker (Enzo Life Sciences) to prevent non-specific background staining. Rabbit monoclonal anti-mouse UCP-1 (Abcam, Cambridge, MA, USA, Cat. ab155117, dilution 1:1000) was used to determine BAT and SAT thermogenesis, whereas F12/80 IgG2a,k antibody (Santa Cruz Biotechnology, Dallas, TX, USA) was used to evaluate macrophage infiltration in SAT. Slides were incubated with the diluted antibodies at 10 µg/mL for 40 min at room temperature. The reaction was developed using a streptavidin biotin reagent + peroxidase (Dako, Glostrup, Denmark) for 15 min, followed by incubation with the peroxidase substrate 3,3′-diaminobenzidine (DAB; SIGMA-Aldrich) for 10 min. Sections were counterstained with hematoxylin, dehydrated in alcohol and xylene, and mounted in resin. Negative controls were incubated with normal human serum diluted 1:100 and a IHC universal negative control reagent (IHC universal negative control reagent, Enzo Life Sciences, Inc., Farmingdale, NY, USA) instead of primary antibody. At least two different sections of each tissue were analyzed. Digital images were obtained from each section at 40× magnification.

### 2.12. Immunoblotting

To evaluate the abundance of selected signaling proteins, samples of frozen skeletal muscle, liver, and brown, subcutaneous and visceral adipose tissues were homogenized at 4 °C in ice-cold RIPA buffer containing phosphate-buffered saline (PBS), 1% NONIDET-P40, 0.5% sodium deoxycholate, 0.1% sodium dodecyl sulphate, 1 mM sodium fluoride, 2 mM sodium orthovanadate, and 1 tablet/10 mL of protease inhibitor mixture (Complete Mini, Roche Diagnostics Corporation, Indianapolis IN, USA) in a TissueLyser (Qiagen, Germantown MD, USA). The samples were incubated on ice for 30 min, centrifuged at 17,400× *g* for 15 min at 4 °C, and the supernatant was transferred to a new tube and stored at −80 °C until use. Protein concentration was determined with the Lowry method. Protein samples (40 μg) were separated on a 10% SDS-polyacrylamide gel and transferred to polyvinylidene difluoride (PVDF) membranes (Hybond-P, Amersham, GE Healthcare, Chicago, IL, USA) using a wet electroblotting System (Bio-Rad, Hercules, CA, USA). The membranes were blocked for 1 h with 5% non-fat dry milk, and incubated with primary antibody diluted in blocking solution overnight. Primary antibodies were as follows: AMPKa 1/2 (Santa Cruz Biotechnology, Dallas, TX, USA, Cat. sc-25792, dilution 1:1250 skeletal muscle), *p*-AMPK, Th2-172 (Santa Cruz Biotechnology, Cat. sc-25792, dilution 1:1250 skeletal muscle), AKT (Merck Millipore, Burlington, MA, USA, Cat. 05-1003), *p*-AKT (Millipore cat SAB5600064 dil 1:1000), UCP-1 (Abcam, Cambridge, MA, USA, Cat. ab155117 dilution 1:1000), GLUT4 (Cell Signaling Technology, Danvers, MA, USA, Cat. 2213), adiponectin (Cell Signaling Technology, Danvers, MA, USA. Cat. 2789) and GAPDH (Abcam, Cat. ab181802, dilution 1:50,000), The membranes were washed three times with TBS-T for 10 min and then incubated with horseradish peroxidase-conjugated secondary antibody (goat anti-rabbit or rabbit anti-goat 1:3500) for 1.5 h. Visualization was performed using a chemiluminescent detection reagent (Millipore). Digital images of the membranes were obtained by a ChemiDoc MP densitometer and processed by Image Lab software (Bio-Rad). The results are reported as a phosphorylated/total protein ratio. A value of 1 was arbitrarily assigned to the control group, which was used as a reference for the other conditions. According to the antibody manufacturer, it is common to find two bands in AKT blots due to posttranslational modifications. Thus, both bands were used in the densitometric analysis. 

### 2.13. Gene Expression Analysis in Liver, Muscle and Adipose Tissue by Quantitative-Polymerase Chain Reaction (PCR)

Total RNA was extracted from liver, skeletal muscle and adipose tissue frozen samples using a Power SYBR Green Cells-to-Ct kit (Invitrogen, Carlsbad, CA, USA). Briefly, frozen tissues were homogenized in ice-cold TRIzol reagent (Invitrogen), in a TissueLyser (Qiagen). Precipitated RNA was resolved by electrophoresis in a 1.5% agarose gel to evaluate integrity. Retro transcription was performed using the RT Master Mix included in the kit, under the following conditions: incubation at 37 °C for 60 min, then 95 °C for 5 min for inactivation of the RT enzyme. Finally, the mRNA abundance of each gene was determined in a LightCycler 480 real-time PCR equipment (Roche) under the following amplification conditions: enzyme activation: 1 cycle at 95 °C for 10 min; PCR: 55 cycles at 95 °C for 15 s, 60 °C 1 min. This was followed by a dissociation curve. The relative abundance of each mRNA was determined for each sample using the ∆∆Cq method and the subtraction of the 36b4 Cq from each sample of Cq. PCR primers. Primer sequences were designed using Primer-BLAST (NCBI, NIH) (Table 2).

### 2.14. DNA Extraction and Preparation of 16S rRNA Gene Amplicon Libraries

After collection, fecal samples were placed in a sterile polypropylene container and immediately transported to the laboratory facilities in ice-filled coolers. Aliquots with 200 mg were made and stored at −80 °C until processing. Bacterial DNA was extracted by using the QIAamp DNA stool kit (QIAGEN, Hilden, Germany) following the manufacturer’s instructions. DNA concentrations were measured by using Nano Drop V3.8.1. The V3–V4 hypervariable region of bacterial 16S rRNA gene was amplified by GeneAmp PCR system 9700 (Applied Biosystems, Waltham, MA, USA). PCR products were purified by using magnetic AMPure XP Beads (Beckman Coulter, Danvers, MA, USA), and then quantified using the Qubit system (Invitrogen, USA) according to the manufacturer’s specifications. A second PCR was applied to the resulting products in which dual indices (containing a 6-nt unique sequence to identify samples when pooled for sequencing) and sequencing adapters were incorporated using the Nextera XT Index kit (Illumina, USA), in order to generate complete libraries. Thereafter, AMPure XP beads were repeated to clean up the library. Finally, the resulting library in each sample was qualified and quantified using an Agilent 4200 TapeStation (Agilent, Santa Clara, CA, USA).

### 2.15. Sequencing and Data Analysis

The 9 libraries were mixed in equimolar concentrations to generate a 4 nM library pool using 10 mM Tris (pH 8.5) as diluent. In addition, libraries were denatured with 0.2 N NaOH and diluted to a final concentration of 10 pM, including a 10% PhiX Control v3 (Illumina, Cat. No. FC-110-3001). The DNA library was sequenced at the Sequencing Unit in the National Institute of Genomic Medicine (INMEGEN) by Illumina Miseq platform (Illumina, San Diego, CA, USA) as described by Caporaso et al. [23]. Illumina FASTQ reads were processed using the QIIME (quantitative insights into microbial ecology) software package. Forward and reverse reads were first merged using join_paired_ends.py script. The resulting sequences were filtered using split_libraries.py script with the following parameters: (r = 3, *p* = 0.75 total read length; q = 3; *n* = 0). The UCHIME algorithm was implemented to safely detect and remove chimeric sequences. Briefly, sequences were clustered into operational taxonomic units (OTUs) using a 97% identity threshold with the UCLUST tool, wrapped within QIIME. Representative sequences were aligned against the Greengenes database, and taxonomy was assigned using the Ribosomal Database Project (RDP) classifier with a minimum support threshold of 80%. The taxonomic composition of the gut microbiota was assessed using METAGENassist. Community diversity was calculated by using the alpha_rarefaction.py script including estimator Chao1 (species richness), Shannon index (species diversity) and species metrics.

### 2.16. Statistical Analysis

All data is expressed as mean values ± SEM. Sample size was selected based on calculations used in our previous published study [22]. Differences between groups were evaluated by one-way ANOVA followed by the Tukey multiple comparison test. Differences were considered significant at *p* ≤ 0.05. Lowercase letters indicate statistical differences between groups. The intervention groups were analyzed by unpaired *t* test. All analysis was performed in GraphPad Prism 7.0 (GraphPad Software, San Diego, CA, USA).

## 3. Results

### 3.1. LC-MS Profiling of Phenolic Extracts

**Figure 1:**
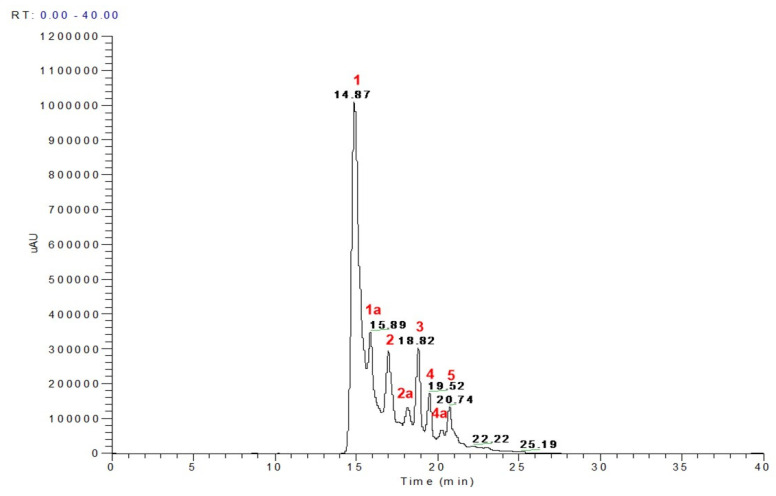
LC chromatogram of Pawnee pecan. Peak assignment 1–5 in red of the identified phenolic compounds by LC-MS are presented in Table 3.

As shown in the LC-MS analysis (Figure 1, Table 3), Procyanidin B2, catechin hexoside, ellagic acid pentoside, methyl ellagic acid hexoside, methyl ellagic acid pentoside, Di-galloyl ellagic acid, ellagic acid galloyl pentoside, and methyl ellagic acid galloyl pentose were identified in Pawnee pecan samples which was similar to phenolics previously identified in the literature for Pawnee pecan [10,11] confirming extracts are formed by condensed tannin and ellagic acid derivatives. Ellagic acid derivatives including ellagitannins are known to be hydrolyzed into ellagic acid and metabolized by the gut microbiota into different types of urolithins. Furthermore, recent work from our group has shown that ellagic acid and urolithins A and B can differentially prevent lipogenesis and inflammation in mature adipocytes while not affecting insulin sensitivity [24].

### 3.2. Consumption of Whole Pecans or a Phenolic Extract of Pecans Prevented Body Weight Gain and Maintained Body Composition of Mice Fed a High-Fat Diet

**Figure 2:**
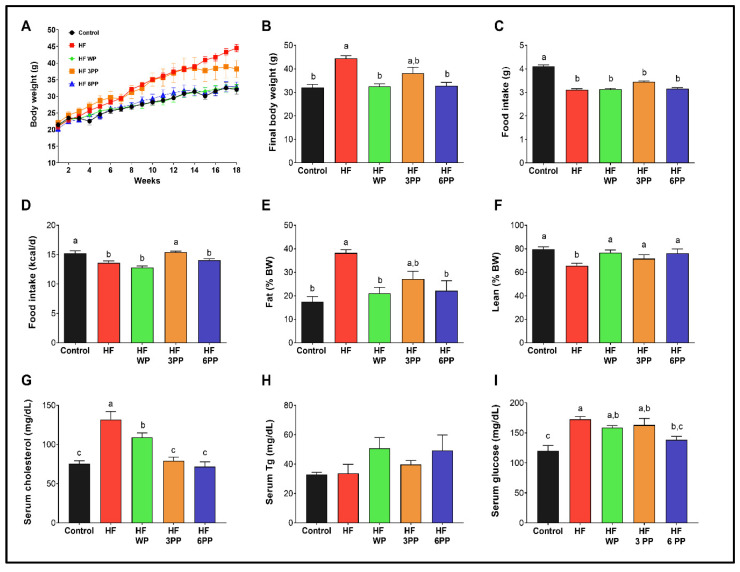
Body weight, food intake expressed as grams (g) or energy (kcal/d), body fat and lean mass content and circulating cholesterol, triglycerides and glucose. (**A**) Body weight change throughout the study, (**B**) body weight at the end of the study, (**C**) food intake in grams/day, (**D**) Energy intake in kcal/day, (**E**) fat mass content (percentage), (**F**) lean mass content (percentage), (**G**) Serum cholesterol, (**H**) Triglycerides and (**I**) Glucose of mice fed the Control diet (Control), a High-fat diet (HF) or an HF diet containing whole pecans (HF WP), 3 mg (HF 3PP) or 6 mg (HF 6PP) of a pecan phenolic extract. Results are presented as the mean ± S.E.M., *n* = 6. Differences were considered statistically significant at *p* < 0.05. Statistical differences between groups are indicated with lowercase letters, where a > b > c.

To evaluate if whole pecans or their phenolic compounds (Table 3) can prevent the appearance of the metabolic alterations associated with a high-fat diet, we fed mice with a high-fat diet (HF), an HF diet containing whole pecans (HF WP) or an HF diet supplemented with 3 mg (HF 3PP) or 6 mg (HF 6PP) of a pecan phenolic extract for 18 weeks compared with mice fed a control diet (Control). As expected, HF mice gained more weight (~37%) than Control mice (~32 g) (Figure 2A,B). Interestingly, HF WP, and HF 6 PP mice gained the same weight as Control mice, and significantly less weight than the HF mice throughout the study (Figure 2A,B), despite similar food and energy intake (Figure 2C,D). The body weight of mice fed with the HF 3PP was similar to that of those fed the HF until week 14, although afterwards it increased at a lower rate (Figure 2A). At the end of the study, the final body weight of mice fed HF 3PP was not significantly different with respect to those fed HF but was also similar to HF WP, HF 6PP and the control mice (Figure 2B). To evaluate if the differences in body weight between groups were due to dissimilar fat mass content, we determined the body composition of each mouse at the end of the 18 weeks. Mice fed the HF diet had a significantly higher percentage of fat mass and lower lean mass content than Control mice (~38 and 63% vs. ~18 and 80%, respectively) (Figure 2E,F). Interestingly, mice fed HF WP and HF 6PP had a significantly lower fat mass content (~44%) and a higher percentage of lean mass than those fed HF, and similar body composition to Control mice (Figure 2E,F). Furthermore, HF mice had a significantly higher circulating cholesterol content (~75%) than Control mice (~75.4 mg/dL) (Figure 2G). The serum cholesterol of mice fed the HF containing whole pecans was marginally lower (~17%) with respect to those fed HF. Notably, consumption of an HF diet supplemented with pecan polyphenols maintained circulating cholesterol levels similar to the control (Figure 2G). The serum triglycerides were not different in any treatment (Figure 2H); however the circulating glucose was elevated in HF mice (~43%) with respect to the control (~120 mg/dL) and only HF 6PP had lower glucose than HF (Figure 2I). These results demonstrate that consumption of whole pecans or a pecan phenolic extract prevents excessive weight and fat mass accretion and the increase in circulating cholesterol induced by a high-fat diet.

### 3.3. Whole Pecans or a Phenolic Extract of Pecans Attenuated Glucose Intolerance and Improved Insulin Sensitivity of Mice Fed a High-Fat Diet

**Figure 3:**
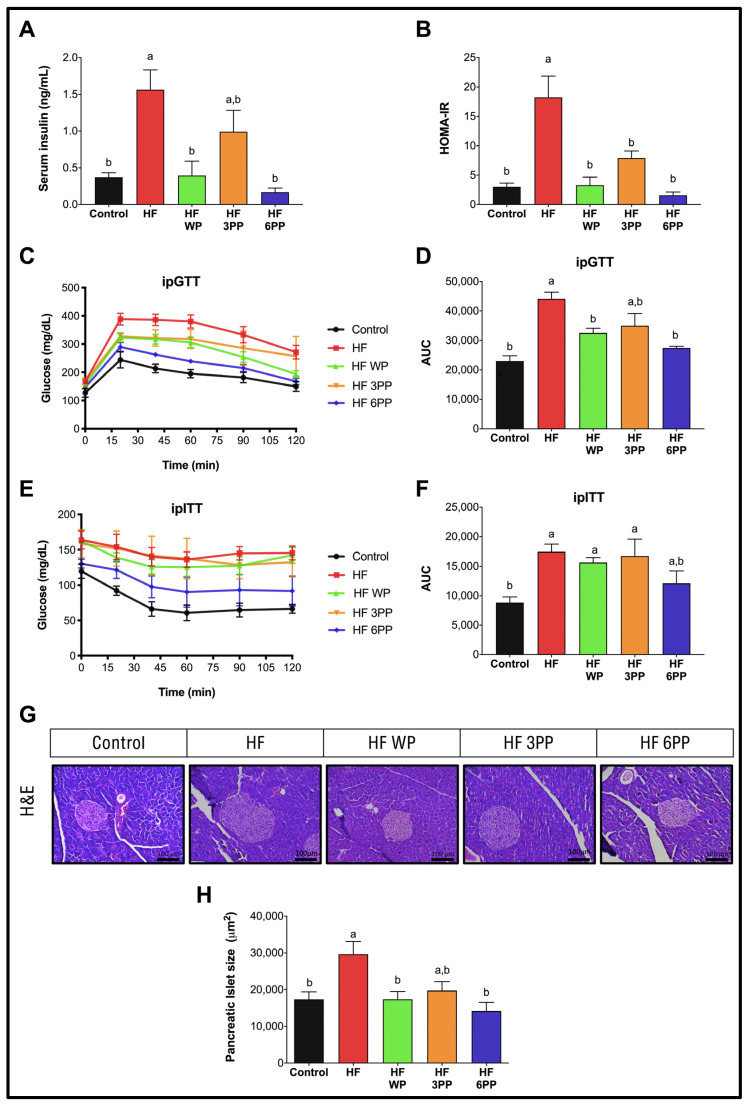
Glucose tolerance, insulin sensitivity and pancreatic islet size. (**A**) Fasting serum glucose, (**B**) Fasting serum insulin, (**C**) Glucose concentrations during intraperitoneal glucose tolerance test (ipGTT), (**D**) ipGTT area under the curve (AUC), (**E**) Glucose concentrations during intraperitoneal insulin tolerance test (ipITT), (**F**) ipITT area under the curve (AUC), (**G**) Islet size quantification and (**H**) Representative hematoxylin and eosin stained pancreatic islets of mice fed the Control diet (Control), a High-fat diet (HF) or a High-fat diet with whole pecans (HF WP), or a High-fat diet with phenolic extract, 3 mg (HF 3PP) or 6 mg (HF 6PP). Results are presented as the mean ± S.E.M., *n* = 6. Differences were considered statistically significant at *p* < 0.05. Statistical differences between groups are indicated with lowercase letters, where a > b.

A common metabolic alteration induced by a high-fat diet is glucose intolerance. Serum insulin was higher in mice fed HF (~320%) than Control mice (Figure 3A). Interestingly, HF WP and HF 6PP mice had significantly lower insulin levels than HF mice and similar to Control mice (~0.373 ng/mL). Thus, despite there being no differences in the fasting serum glucose, the HOMA-IR index (Figure 3B) of mice fed HF WP, HF 3PP and HF 6PP was lower (~82, 56, and 91%, respectively) than HF mice, suggesting an improvement in glucose metabolism. To confirm the previous observations, mice were subjected to intraperitoneal glucose and insulin tolerance tests (ipGTT and ipITT, respectively). During the ipGTT, mice fed the HF diet had a significantly higher glucose area under the curve (AUC) (~92%) than Control mice (Figure 3C,D). Notably, mice fed HF WP and HF 6PP had a significantly lower glucose AUC (~26 and 37%, respectively) than HF mice and similar to Control mice. The AUC from mice fed HF 3PP presented a trend lower than that from HF; however it was not significantly different. (Figure 3C,D). In line with the ipGTT results, HF mice were less insulin-sensitive than Control mice during the ipITT test showing HF to have a higher AUC (~107%) (Figure 3E,F). As observed during the ipGTT, only HF 6PP mice had an improvement in insulin sensitivity that was similar to the Control mice. These results indicate that the pecan phenolic extract improves glucose tolerance in a dose-dependent manner. Hyperinsulinemia is frequently associated with pancreatic islet hypertrophy. Accordingly, we found a direct association between circulating insulin and pancreatic islet size in which HF mice presented a significant increase in pancreatic islet size (~71%) with respect to Control mice (Figure 3G,H). Interestingly, mice fed HF supplemented with whole pecans or a pecan polyphenol extract had significant lower pancreatic islet size than HF mice and similar to Control mice (~17,344 μm^2^). These results indicate that consumption of whole pecans or a phenolic extract of pecans modulates glucose metabolism and pancreatic islet morphology in mice fed a high-fat diet.

### 3.4. Consumption of Whole Pecans or a Phenolic Extract of Pecans Increase Energy Expenditure in Mice Fed a High-Fat Diet

**Figure 4:**
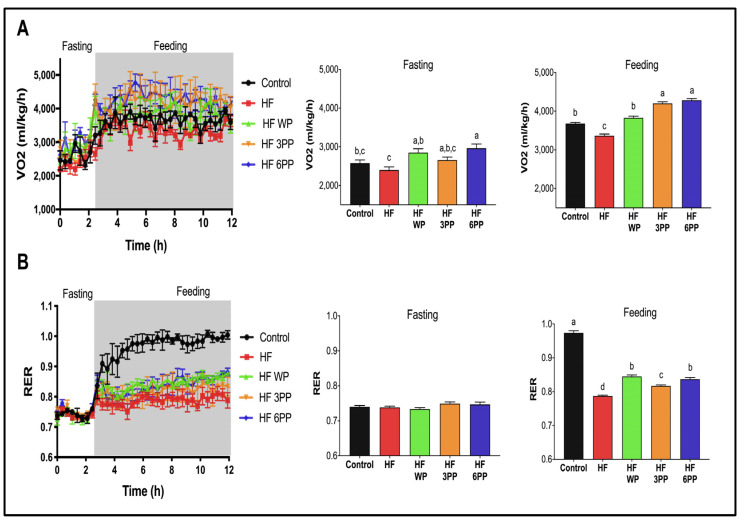
Whole-body energy expenditure and substrate oxidation. (**A**) Oxygen consumption (VO_2_) during fasting and feeding periods. Clear and grey zones indicate the fasting and feeding periods, respectively. (**B**) Respiratory exchange ratio (RER) and average RER during fasting and feeding periods of mice fed the Control diet (Control), a High-fat diet (HF), a High-fat diet with whole pecans (HF WP), or a High-fat diet with phenolic extract, 3 mg (HF 3PP) or 6 mg (HF 6PP). Results are presented as the mean ± S.E.M., *n* = 6. Differences were considered statistically significant at *p* < 0.05. Statistical differences between groups are indicated with lowercase letters, where a > b > c > d.

To determine if the differences in body weight, fat mass, glucose tolerance and insulin sensitivity of HF WP and HF 6PP with respect to HF mice were associated with changes in energy expenditure, we performed an indirect calorimetry during fasting and feeding states after 18 weeks on the experimental diets. First, HF mice consumed 10% less O_2_ than Control mice (~3679 mL/kg/h) during the fed state (Figure 4A). Interestingly, HF WP mice had similar O_2_ consumption to Control mice. Regarding HF 3PP and HF 6PP, both had significantly higher O_2_ consumption than both Control (~14–16%) and HF mice (~24–27%). We then assessed the type of metabolic substrate used, based on the respiratory exchange ratio (RER). There were no significant differences among groups during fasting (RER 0.76–0.79), indicating that the animals were obtaining their energy primarily from fatty acids (Figure 4B). However, during the feeding state, the RER of Control mice increased to 0.98, whereas HF mice increased to only 0.79, indicating the development of metabolic inflexibility. Mice fed HF WP, HF 3PP, and HF 6PP had significantly higher postprandial RER (0.84, 0.81, 0.83, respectively) than those fed HF, indicating increased glucose utilization as an energy substrate. These results demonstrated that whole pecans or a pecan phenolic extract increases energy expenditure and metabolic flexibility in mice despite the elevated fat content of the diet.

### 3.5. Increased Brown Adipose Tissue Thermogenic Activity in Mice Fed a High-Fat Diet Supplemented with Whole Pecans or a Pecan Phenolic Extract

**Figure 5:**
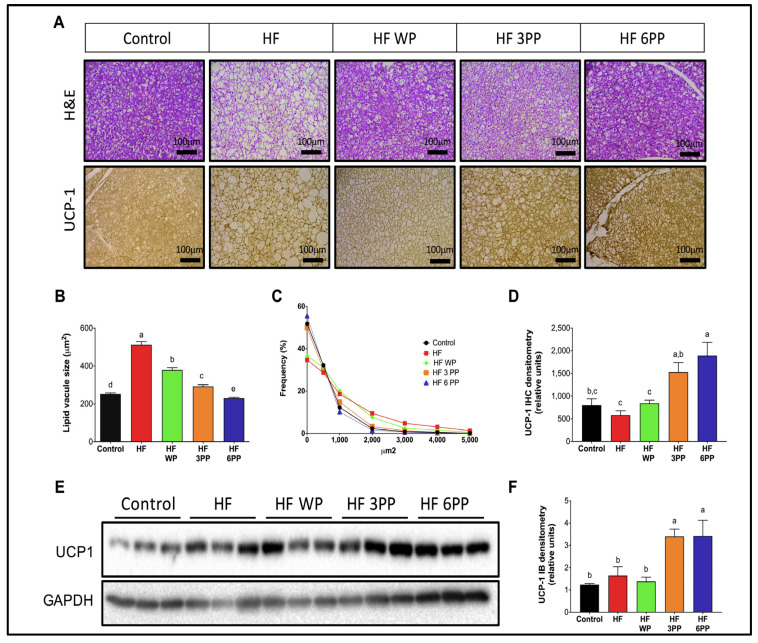
Brown adipose tissue morphology, immunostaining and immunoblotting in mice. (**A**) Hematoxylin & eosin staining and uncoupling protein 1 (UCP-1) in brown adipose tissue (BAT), (**B**) Brown adipose tissue vacuole area, (**C**) Frequency distribution of vacuole areas in BAT, (**D**) Immunostaining densitometry, (**E**) Immunoblot of UCP-1 and (**F**) Abundance relative of UCP-1 in BAT of mice fed the Control diet (Control), a High-fat diet (HF) or a High-fat diet with whole pecans (HF WP), or a High-fat diet with phenolic extract, 3 mg (HF 3PP) or 6 mg (HF 6PP). Results are presented as the mean ± S.E.M., *n* = 6. Differences were considered statistically significant at *p* < 0.05. Statistical differences between groups are indicated with lowercase letters, where a > b > c > d > e.

To evaluate the participation of brown adipose tissue (BAT) thermogenesis in energy expenditure of mice fed an HF diet, we evaluated BAT morphology and uncoupling protein 1 (UCP-1) abundance. Hematoxylin and eosin staining revealed numerous enlarged vacuoles in brown adipocytes of the HF group (Figure 5A), a morphological feature of BAT dysfunction [25]. Conversely, brown adipocytes from mice fed HF WP, HF 3PP or HF 6PP presented a morphology similar to those from Control mice. Automated morphometric evaluation showed that the mean vacuole size of mice fed HF was significantly higher (~103%) than control (~252 µm^2^) and from all other groups (Figure 5B), indicating impaired substrate utilization. Interestingly, the mean BAT vacuole area of mice fed whole pecans or pecan polyphenol extract was lower than HF mice, indicating an enhanced oxidative capacity. Remarkably, the mean vacuole area of HF 6PP mice was even lower than Control mice. Accordingly, the frequency distribution analysis of vacuole areas of BAT indicates that more than 50% of vacuoles from Control, HF 3PP and HF 6PP mice were below 250 µm^2^ (Figure 5C), whereas 10% of vacuoles from HF mice were larger than 2000 µm^2^. Brown adipose tissue oxidizes energy substrates and releases heat through the activity of the mitochondrial oxidative phosphorylation-uncoupler UCP-1. As revealed by immunostaining and immunoblot, UCP-1 abundance in BAT from HF mice was not different to that in Control mice (Figure 5A,D,E). Mice fed HF WP had similar UCP-1 expression to that of HF or Control mice. However, UCP-1 protein content in mice fed HF 3PP or HF 6PP was higher than in all other groups. These results indicate that a pecan polyphenol extract increases brown adipocytes oxidative metabolism and energy expenditure, preventing BAT dysfunction and excessive fat accumulation.

### 3.6. Whole Pecans or a Pecan Phenolic Extract Intake Increases Mitochondrial Content and Reduced Lipid Accumulation in Skeletal Muscle of Mice Fed a High-Fat Diet

**Figure 6:**
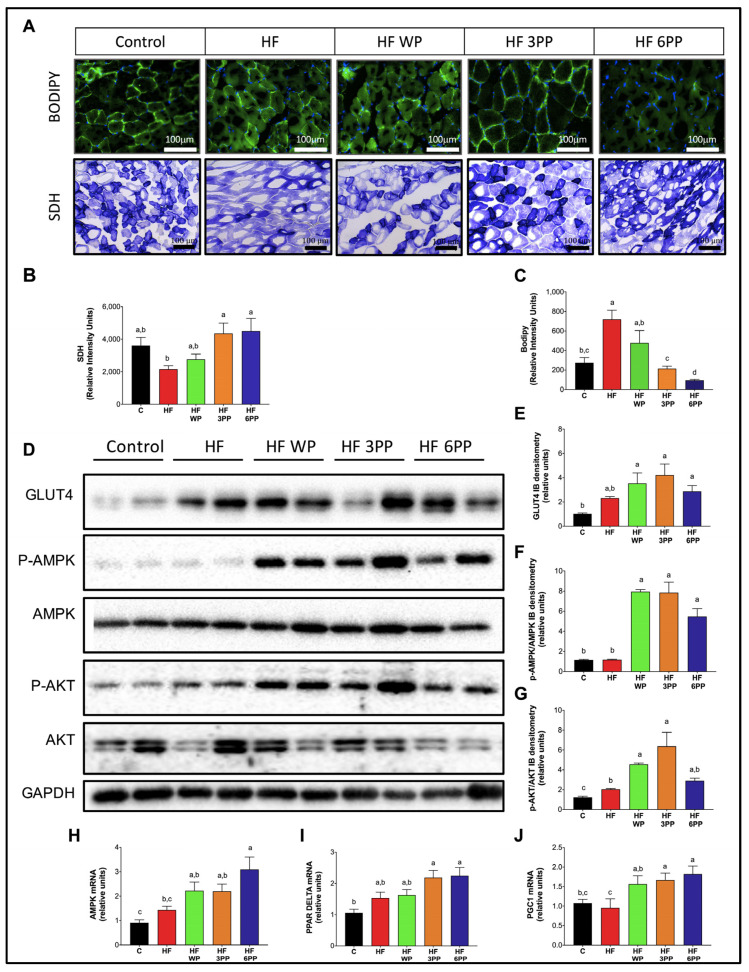
Mitochondrial activity and lipid and glucose metabolism in skeletal muscle. (**A**) Skeletal muscle cryosections stained with BODIPY and succinate dehydrogenase-nitroblue tetrazolium (SDH), (**B**) Densitometric SDH staining quantification, (**C**) Densitometric BODIPY staining quantification, (**D**) GLUT4, phosphor-AMPK, total AMPK; phospho-AKT, total AKT, and GAPDH immunoblot, (**E**) GLUT4 densitometric quantification, (**F**) Densitometric quantification of *p*-AMPK/AMPK and (**G**) *p*-AKT/AKT ratio, (**H**) AMPK mRNA, (**I**) PPAR delta mRNA and (**J**) PGC-1 alpha abundance in skeletal muscle of mice fed the Control diet (Control), a High-fat diet (HF) or an HF diet containing whole pecans (HF WP), 3 mg (HF 3PP) or 6 mg (HF 6PP) of a pecan phenolic extract. Results are presented as the mean ± S.E.M., *n* = 6. Differences were considered statistically significant at *p* < 0.05. Statistical differences between groups are indicated with lowercase letters, where a > b > c > d.

Skeletal muscle is the largest organ in the body and the most important contributor to basal metabolic rate and energy expenditure. However, increased fatty acid availability and reduced mitochondrial activity during obesity leads to intramyocellular lipid accumulation and glucose intolerance [26]. To evaluate the effects of whole pecans and pecan polyphenol extracts in skeletal muscle glucose and lipid metabolism, we assessed mitochondrial activity and lipid content in skeletal muscle cryosections through succinate dehydrogenase (SDH) and BODIPY staining, respectively. As observed in Figure 6A, mice fed HF had lower SDH staining (~40%) than controls, indicating reduced mitochondrial activity. Skeletal muscle from HF WP also had a lower mitochondrial content (~23%) than controls. However, mice fed HF 3PP or HF 6PP presented higher mitochondrial content than those fed HF and even Control mice (~108 and 20% higher, respectively). Accordingly, the skeletal muscle of HF and HF WP mice had a higher intramyocellular lipid content with respect to controls (Figure 6A–C). Conversely, HF 3PP or HF 6PP mice had significantly fewer lipid infiltrates in muscle than HF or HF WP mice. To evaluate if the reduction in lipid accumulation in the skeletal muscle of mice fed pecan polyphenols is associated with improved insulin signaling and glucose transport, we measured AKT phosphorylation and GLUT4 content in skeletal muscle protein homogenates. Mice fed HF had lower phospho-AKT like Control; mice fed HF had similar measured GLUT4 with respect to Control group mice (Figure 6D–G). Remarkably, mice fed whole pecans or pecan polyphenol extract had higher phosphorylated AKT and GLUT4 abundance with respect to HF and Control mice. To gain insight into the mechanisms involved in the beneficial effects of pecan phenolics on skeletal muscle metabolism, we measured AMPK, PGC-1 alpha and PPAR delta mRNA abundance in skeletal muscle. As observed in Figure 6H–J, HF WP, HF 3PP and HF 6PP mice showed increased gene expression of AMPK, PGC-1 alpha and PPAR delta in skeletal muscle with respect to HF and Control mice. These results indicate that pecan polyphenols increase mitochondrial activity in skeletal muscle by stimulating PPAR delta-mediated gene expression.

### 3.7. Whole Pecans or a Pecan Phenolic Extract Prevents Adipose Tissue Dysfunction in Mice Fed a High-Fat Diet

**Figure 7:**
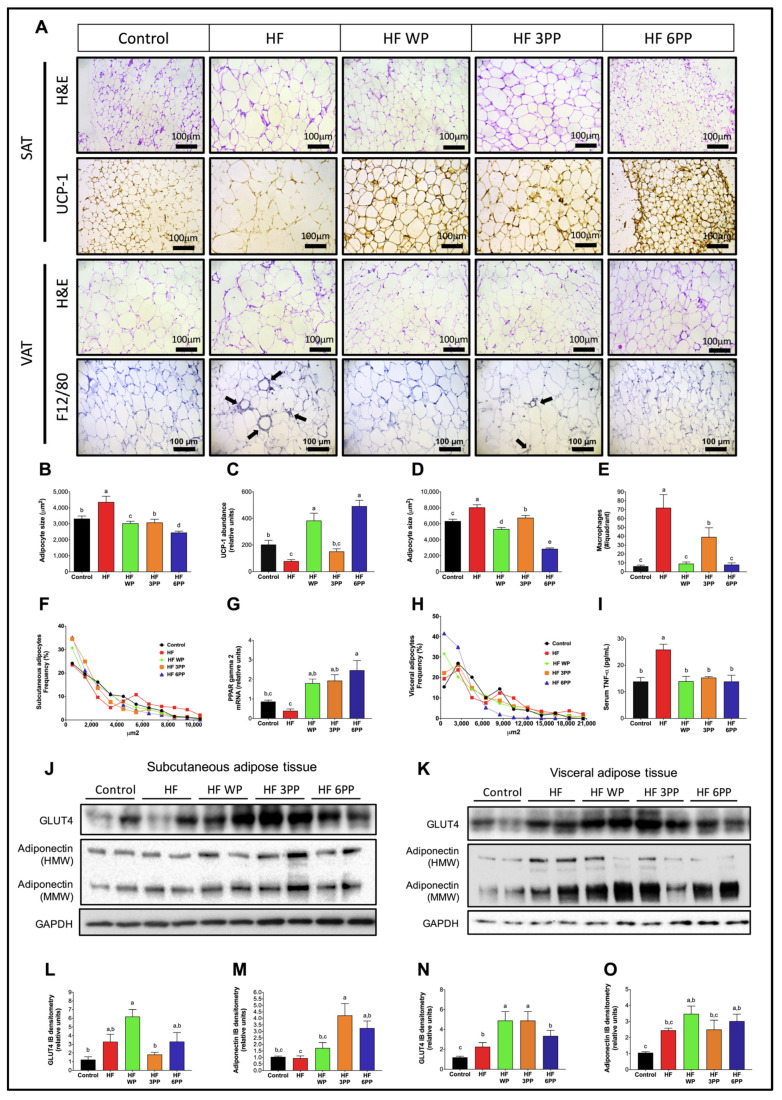
Subcutaneous (SAT) and visceral (VAT) adipocyte tissue morphology H&E, UCP-1 and F4/80 immunostaining. Arrows mark macrophages around dead adipocytes (**A**), SAT adipocyte size (**B**), UCP-1 relative abundance (**C**), VAT adipocyte size (**D**), F4/80 immunostaining content (**E**), SAT frequency percentage (**F**), PPAR-gamma 2 mRNA relative units (**G**), VAT frequency percentage (**H**), serum TNF-α (**I**), SAT and VAT immunoblot (**J**,**K**), SAT GLUT4 and adiponectin densitometry (**L**,**M**) and VAT GLUT4 and adiponectin densitometry (**N**,**O**) in mice fed the Control diet (Control), a High-fat diet (HF) or an HF diet containing whole pecans (HF WP), 3 mg (HF 3PP) or 6 mg (HF 6PP) of a pecan phenolic extract. Results are presented as the mean ± S.E.M., *n* = 6. Differences were considered statistically significant at *p* < 0.05. Statistical differences between groups are indicated with lowercase letters, where a > b > c > d > e.

Fat accumulation in response to high-energy diets causes adipocyte dysfunction characterized by adipocyte hypertrophy. To evaluate adipocyte hypertrophy, we performed automated morphometric analyses in inguinal/subcutaneous and retroperitoneal/visceral adipose tissues (SAT and VAT, respectively). As observed in Figure 7A,B,D, hematoxylin & eosin-stained adipocytes from SAT and VAT of HF mice were significantly larger than those from Control mice. Notably, the mean adipocyte size of SAT and VAT of mice fed whole pecans or pecan polyphenol extract was significantly lower than that of HF mice. The reduction in adipocyte size was strikingly evident in mice fed HF 6PP whose mean adipocyte area was significantly lower than that of Control mice in both adipose tissue depots. The area frequency distribution of adipocytes in SAT and VAT in mice fed the different diets also presented clear differences. Subcutaneous adipose tissue from HF mice had a higher proportion of adipocytes above 7000 µm^2^ with respect to Control and the pecan or polyphenol-fed mice (Figure 7F). Adipocyte distribution in SAT of mice fed HF WP, HF 3PP or HF 6PP presented a higher proportion of adipocytes in the range below 4500 µm^2^. This trend was similar to that in VAT, where adipocytes from HF mice were mainly above 5000 µm^2^, whereas those from Control, HF WP and HF 3PP mice were smaller than 5000 µm^2^ (Figure 7H). Interestingly, adipocyte size distribution of VAT from HF 6PP mice presented a higher proportion of small adipocytes (<2000 µm^2^) than any other group. Adipocyte size is dependent on the proliferative capacity of adipose tissue. In response to increased energy storing demands, adipose tissue increases pre-adipocyte recruitment and differentiation into mature adipocytes favoring a hyperplasic expansion. However, impaired recruitment and differentiation of new adipocytes leads to hypertrophy of existing adipocytes, leading to reduced thermogenic capacity, adipocyte death, macrophage recruitment and impaired glucose uptake [27,28,29]. Accordingly, SAT from HF mice had lower UCP-1 abundance (~61%) with respect to that from Control mice (Figure 7A,C). Interestingly, mice fed HF WP and HF 6PP had higher thermogenic activity than all other groups as assessed by UCP-1 content. The increase in UCP-1-mediated thermogenesis in SAT could also account for the increased VO_2_ consumption in these groups. Moreover, macrophage recruitment, as assessed by F4/80 immunostaining was evident in VAT from mice fed HF (Figure 7A,E). Mice fed HF 3PP had significantly fewer macrophage infiltrates than those fed HF. We did not find macrophage infiltrates in VAT from control, HF WP or HF 6PP groups. M1 macrophage infiltrate in adipose tissue increases the synthesis and release of pro-inflammatory cytokines, such as TNF-alpha, favoring the development of a low-grade systemic inflammatory profile. Accordingly, consumption of an HF diet markedly increased TNF-alpha in serum (~86%) with respect to Control mice (~13.9 pg/mL), while TNF-alpha concentration in the serum of mice fed HF WF, HF 3PP or HF 6PP was similar to that of those fed the Control diet (Figure 7I). Furthermore, the adipogenic transcription factor PPAR- gamma 2 was higher in mice fed with HF WP, HF 3PP and HP 6PP compared to HF mice (Figure 7G). To evaluate the capacity for insulin-mediated glucose uptake in SAT and VAT, we evaluated GLUT4 protein abundance in adipose tissues. As observed in Figure 7J,K,L,N, mice fed HF WP, HF 3PP or HF 6PP had a higher GLUT4 content in SAT and VAT with respect to HF and control mice, indicating increased glucose uptake capacity. Adiponectin was also more abundant in both adipose tissues of mice fed whole pecans and the pecan polyphenols extract (Figure 7J,K,M,O). These results indicate that supplementation with whole pecans or a pecan polyphenol extract increases adipocyte differentiation, preventing adipocyte hypertrophy and death, macrophage recruitment and pro-inflammatory cytokine release that compromise the main metabolic and functional abnormalities observed in adipose tissue in response to an HF diet.

### 3.8. Consumption of Whole Pecans or a Phenolic Extract of Pecans Prevented Hepatic Steatosis and Metabolic Signaling Alterations in Liver of Mice Fed a High-Fat Diet

**Figure 8:**
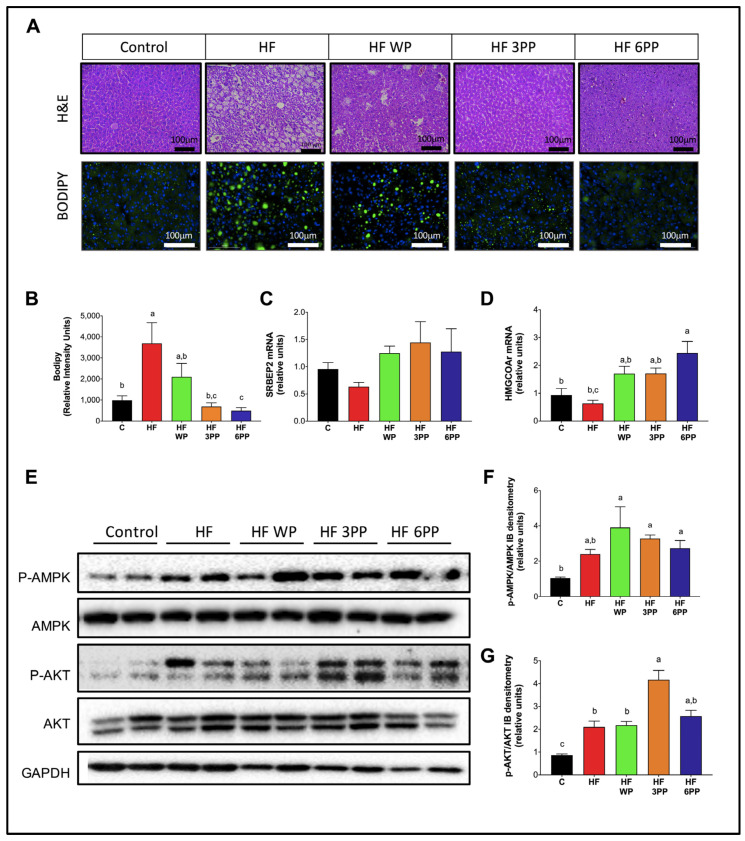
Hepatic histomorphology H&E or BODIPY staining (**A**), BODIPY densitometric (**B**), SREBP-2 and HMGCoA mRNA relative units (**C**,**D**), phospho-AMPK, total AMPK; phospho-AKT, total AKT, and GAPDH immunoblot (**E**), Densitometric quantification of *p*-AMPK/AMPK ratio (**F**) and Densitometric quantification of *p*-AKT/AKT ratio (**G**) of mice fed the Control diet (Control), a High-fat diet (HF) or an HF diet containing whole pecans (HF WP), 3 mg (HF 3PP) or 6 mg (HF 6PP) of a pecan phenolic extract. Results are presented as the mean ± S.E.M., *n* = 6. Differences were considered statistically significant at *p* < 0.05. Statistical differences between groups are indicated with lowercase letters, where a > b > c.

Adipose tissue dysfunction has an important impact in hepatic metabolism. Hypertrophic visceral adipocytes release a high amount of free fatty acids and pro-inflammatory cytokines to the liver, leading to fat accumulation and impairment of metabolic signaling. To assess hepatic histomorphology and lipid content, we performed H&E or BODIPY staining in liver. As expected, mice fed HF had overt steatosis as revealed by the numerous lipid vacuoles (Figure 8A,B). Interestingly, mice fed with whole pecans or the pecan polyphenol extract presented fewer lipid vacuoles and thus a lower BODIPY densitometric signal than HF mice. Phosphorylated AMPK and AKT abundance was higher in mice fed with whole pecans and HF 3 PP, respectively, in the liver of mice fed with HF (Figure 8E–G). In the liver, the transcription factor SREBP2 is activated when the cholesterol content in endoplasmic reticulum membranes is reduced [30]. To determine hepatic cholesterol metabolism, we evaluated the mRNA abundance of SREBP2 and one of its target genes; the enzyme HMGCoA reductase, involved in cholesterol synthesis. As observed in Figure 8C,D, the SREBP2 mRNA content in the livers of mice fed HF WP, HF 3PP and HF 6PP was higher (~2.7-, 3.3- and 2.9-fold change, respectively) with respect to HF and similar to Control mice. Accordingly, HMGCoA reductase mRNA was augmented in the livers of mice fed HF WP, HF 3PP and HF 6PP (~2.7-, 2.7- and 3.8-fold change, respectively) compared to HF and Control mice. These results indicate that whole pecans or a pecan polyphenol extract prevent hepatic steatosis, and increase cholesterol mobilization in mice fed an HF diet.

### 3.9. Whole Pecans and a Phenolic Extract of Pecans Improves the Gut Microbiota Induced by a High-Fat Diet in Mice

**Figure 9:**
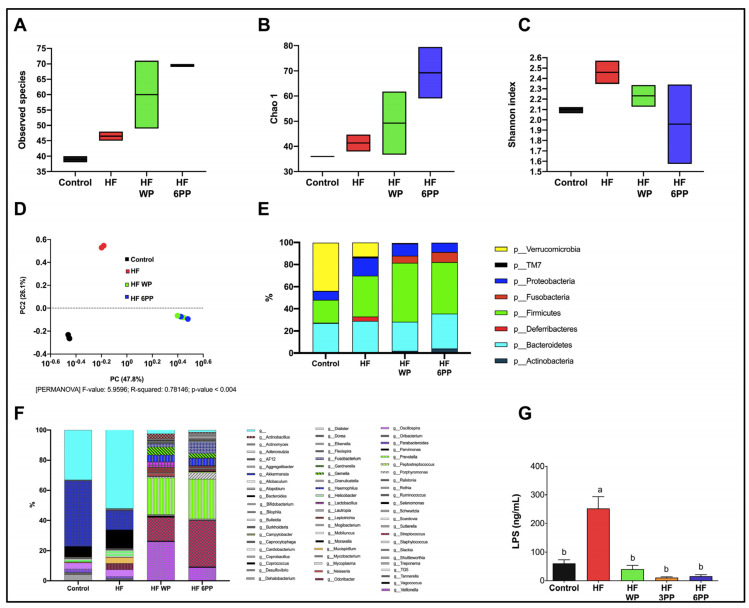
Whole pecans and phenolic extract of pecans improves the gut microbiota. (**A**) Observed species, (**B**) Chao1, (**C**) Shannon index, (**D**) Beta-diversity, (**E**) Phyla diversity, (**F**) Taxonomy level and (**G**) Serum LPS in mice fed the Control diet (Control), a High-fat diet (HF) or a High-fat diet with whole pecans (HF WP), or a High-fat diet with phenolic extract, 3 mg (HF 3PP) or 6 mg (HF 6PP). Results are presented as the mean ± S.E.M., *n* = 6 mice per group and analyzed by one-way ANOVA followed by the Tukey multiple comparison post hoc test. The differences were considered statistically significant at *p* < 0.05. Mean values with different lowercase letters show statistical differences between each other, where a > b.

High energy diets exert profound changes in intestinal microbial communities, leading to dysbiosis, increased lipopolysaccharide (LPS) release and systemic low-grade inflammation. To evaluate whether components of whole pecans or a pecan polyphenol extract can act as prebiotics, we evaluated fecal microbial diversity. Total RNA was isolated from fecal samples collected at the end of the study. From the Illumina 300 bp paired-end sequencing of the amplicon targeting the V3–V4 region of 16S rRNA gene, we generated a total of 580,297 high-quality sequences from the fecal samples with an average of 64,477 sequences per sample. The “non-phylogeny-based” metrics (Observed species, Chao1 and Shannon index) were used to describe alpha diversity (Figure 9A–C). Supplementation of the diet with whole pecans or the 6 mg phenolic extract increased observed species and richness. Regarding beta-diversity (Figure 9D), the separation among the groups was due to the pecan content in the diet (PC1 = 47.6% and PC2 = 25%; PERMANOVA: F-value: 5.8528; R-squared: 0.77835; *p*-value < 0.004). A total of eight phyla were detected. *Firmicutes* and *Bacteroidetes* were the predominant phyla, followed by *Verrucomicrobia*, *Proteobacteria*, *Fusobacteria*, *Actinobacteria* and *Deferribacteres* which were less abundant (Figure 9E). In order to determine whether any taxa at different taxonomic levels were enriched among groups we performed an analysis using the LEfSe algorithm (Figure 9F). There were no significant differences among the groups at any level (phyla, class, order, family or genus). To determine if the increase in microbiota diversity observed in mice fed whole pecans or the polyphenol extracts is associated with reduced circulating LPS, we assessed serum LPS concentration. As observed in Figure 9G, serum LPS in HF mice was higher (~316%, *p* < 0.05) than Control mice (~60 ng/mL), whereas that from HF WP, HF 3PP and HF 6PP mice was significantly lower (~83, 95 and 93%, respectively) than HF and similar to Control mice. These results indicate that pecan phenolics shapes intestinal microbial communities, maintaining eubiosis despite the elevated fat content in the diet.

In the present study, the HF group showed significantly reduced numbers of observed species compared with those in the pecans-supplemented group. The polyphenol content of pecans is likely responsible for the greater alpha-diversity. Sustained consumption of polyphenol-rich diets can stimulate the growth of beneficial bacteria [31,32], thereby promoting greater diversity as well.

Furthermore, whole pecan (WP) or pecan polyphenols (PP) can explain, at most, 73.9% of the variance in beta-diversity. Increased microbiome diversity could promote higher stability of the microbiome in the long term, thereby contributing to functional resilience against extreme stress and perturbations as purported by the classical ecological resilience theory [33]. Differences in the relative abundance of specific bacterial taxa have been observed with almond, pistachio, and walnut consumption over short periods [34,35,36].

### 3.10. A Dietary Intervention with a Pecan Phenolic Extract Improves Metabolic Alterations of Obese Mice

**Figure 10:**
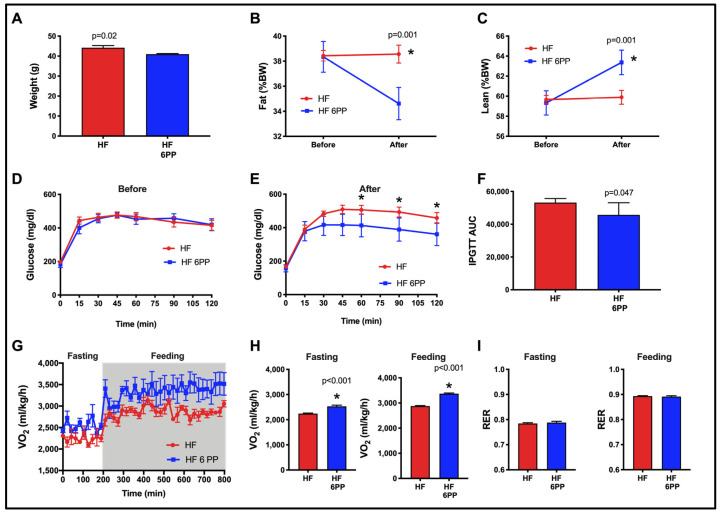
Therapeutic potential of a phenolic extract of pecans on High-fat diet. (**A**) Body weight, (**B**) Fast mass, (**C**) Lean mass, (**D**) glucose tolerance before intervention with HF 6PP diet, (**E**) glucose tolerance after intervention with HF 6PP diet, (**F**) AUC glucose after intervention with HF 6PP diet, (**G**) Energy expenditure, (**H**) Average VO_2_ during fasting and feeding periods, (**I**) Average RER during fasting and feeding periods. High-fat diet and High-fat diet with phenolic extract 6 mg (HF 6PP). Results are presented as the mean ± S.E.M., *n* = 6 mice per group and analyzed by unpaired *t* test. The differences were considered statistically significant at *p* < 0.05 and denoted with an *.

Given that HF 6PP was able to prevent body weight and fat mass gain, glucose intolerance, insulin resistance and improved energy expenditure and gut microbiota diversity, we finally evaluated whether the phenolic extract of pecans had a therapeutic potential. Thus, we performed an intervention study in mice that had previously been fed a high-fat diet for 18 weeks, and presented increased body weight, fat mass, and glucose intolerance. Notably, consumption of HF 6PP for 30 days significantly reduced body weight (Figure 10A) and fat mass (Figure 10B), and increased lean mass (Figure 10C), glucose tolerance (Figure 10D,E) AUC glucose (Figure 10F) and energy expenditure (Figure 10G–I). Altogether, these results demonstrate that phenolic extract of pecans improves the metabolic derangements induced by a high-fat diet.

## 4. Discussion

Pecans are considered functional foods due to their high content of nutraceuticals such as unsaturated fatty acids, dietary fiber and polyphenols. Basic and clinical studies have demonstrated the beneficial effects of different foods rich in nutraceuticals in the prevention of obesity and metabolic syndrome. In the present study, using mice fed a high-fat diet we demonstrated that whole pecans (*Carya illioinensis*) or a pecan phenolic extract (mainly condensed tannin and ellagic acid derivatives) prevented obesity, liver steatosis and diabetes by reducing dysbiosis, inflammation, and increasing skeletal muscle mitochondrial content and whole-body energy expenditure. In order to identify the early targets of pecan polyphenols that prevents the development of the biochemical and molecular alterations in metabolic organs of mice fed an HF diet, we first defined a framework for the progression of metabolic disorders during diet-induced obesity as early and late events.

### 4.1. Proposed Model of Progression of HF Diet Mediated Metabolic Disorder Based on Early and Late Events

**Figure 11:**
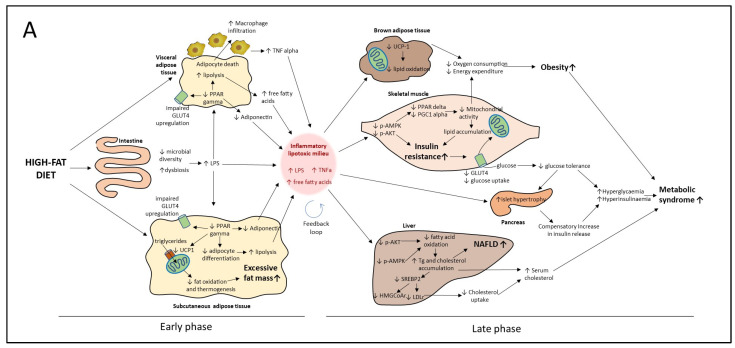

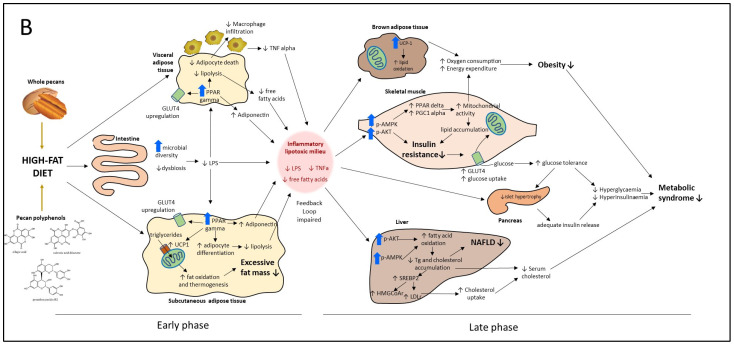
Proposed model of progression of HF diet-mediated metabolic disorder based on early and late phase events (**A**). Molecular targets of whole pecan and pecan phenolic extracts in preventive and intervention strategies (**B**). Vertical black arrows in A and B indicate up- or down-regulation events, while in B vertical blue arrows indicate molecular target direct effects of pecan phenolic bioactive compounds while vertical black arrows indicate indirect effects.

The alterations in metabolic homeostasis induced by high-fat diets can be viewed as a series of interconnected events that synergize with each other, leading to the development of chronic diseases. However, these alterations do not develop simultaneously but rather in a sequential order. Cumulative evidence indicates that the metabolic alterations leading to NAFLD, type 2 diabetes and cardiovascular disease can be divided into early and late events with respect to the onset of the alterations in each organ or tissue. In Figure 11A we propose a model of metabolic alterations divided into early and late responses due to a high-fat diet intake, based on previous reports and results of the present study. Accordingly, early alterations in response to high-energy diets includes the reduction in colonic microbial diversity that leads to dysbiosis and the release of pro-inflammatory bacterial-derived endotoxins such as lipopolysaccharide (LPS) [37] (Figure 9A–G). Surplus energy is stored in subcutaneous and visceral adipose tissues (SAT and VAT, respectively) through a PPAR gamma-dependent increase in adipocyte recruitment and differentiation. The nuclear receptor PPAR gamma also induces in situ fat oxidation and thermogenesis in SAT to prevent excessive lipid accumulation [38]. However, chronic overnutrition reduces PPAR gamma activity, preventing further adipose tissue expansion, which leads to adipocyte hypertrophy and dysfunction (Figure 7A–O). Dysfunctional adipocytes are characterized by reduced adiponectin synthesis and secretion, unrestrained lipolysis and adipocyte death [39]. Adipocyte death induces macrophage infiltration which in turn synthesizes and release pro-inflammatory cytokines such as TNF alpha (Figure 7I) Increased local TNF alpha along with systemic LPS exacerbates adipose tissue dysfunction through reduction in PPAR gamma transcriptional activity [40]. Together, the consequences of intestinal dysbiosis and adipose tissue dysfunction are the increase in circulating LPS, TNF alpha and free fatty acids along with a reduction in the anti-inflammatory adipokine adiponectin (Figure 11A).

These early alterations induce a pro-inflammatory and lipotoxic milieu prompting the later alterations in peripheral organs such as liver, skeletal muscle, brown adipose tissue and pancreas. We propose that this early pro-inflammatory and lipotoxic milieu corresponds to a low-grade inflammation state that through a feedback loop mechanism present in most tissues derives into a chronic inflammation state prompting and sustaining the late events described below.

In the liver, free fatty acids accumulate as triglycerides leading to hepatic steatosis (Figure 8A,B). Lipid accumulation along with pro-inflammatory cytokines and endotoxins impairs hepatic insulin signaling and energy sensing by a reduction in AKT and AMPK activity respectively preventing the up-regulation of fat-oxidizing enzymes [41]. Increased lipid accumulation in liver generates a disbalance in the cholesterol metabolism causing the retention of the transcription factor SREBP-2 in the endoplasmic reticulum and, thus, the reduction in SREBP-2-mediated gene expression [42]. SREBP-2 regulates circulating cholesterol by the up-regulation of lipoprotein receptors and, thus, a reduction in SREBP-2 provokes an increase in cholesterol-rich lipoproteins [30] (Figure 2G).

In skeletal muscle, free fatty acids, LPS and TNF alpha also reduce AKT and AMPK activity. In skeletal myocytes, AKT is the main mediator in insulin-induced GLUT4 translocation to the plasma membrane. A reduction in AKT phosphorylation causes insulin resistance, leading to a reduction in glucose uptake [43] (Figure 3B–F). Since skeletal muscle accounts for 40% of postprandial glucose uptake, a reduction in glucose uptake in this tissue favors hyperglycemia (Figure 2I). Increased skeletal-muscle insulin resistance stimulates a compensatory increase in pancreatic insulin secretion, causing beta cell hypertrophy and hyperinsulinemia [44] (Figure 3A,G,H). In advanced stages of the metabolic syndrome, the pro-inflammatory and lipotoxic milieu in the pancreas causes beta cell death and overt diabetes [45].

AMPK activity in skeletal muscle stimulates PPAR delta and PGC-1a-mediated mitochondrial gene transcription. Inflammatory signaling as well as lipotoxicity impair AMPK signaling in skeletal muscle, decreasing mitochondrial biogenesis and consequently leading to a reduction in glucose and lipid oxidation [46] (Figure 6A,B). The decline in mitochondrial oxidative capacity favors fatty acid accumulation in skeletal myocytes, exacerbating insulin resistance [47] (Figure 6A,C). Impaired mitochondrial function in skeletal muscle also reduces whole-body energy expenditure, prompting fat accumulation (Figure 4). Brown adipose tissue also participates in energy balance through UCP-1-mediated thermogenesis. However, inflammatory cytokines and lipotoxicity reduce UCP-1 activity and thermogenesis in brown adipocytes [48] (Figure 5A,D). The coupled reduction in skeletal muscle and brown adipose tissue energy expenditure and oxygen consumption induces a positive energy balance and obesity (Figure 2A,B,E and Figure 4). To our knowledge, this is the first integrative model describing the metabolic dysfunction in a holistic approach of early and late events induced by an HF diet. Its importance lies in its potential use in the design of preventive and therapeutic strategies as well as to identify molecular targets for bioactive nutraceuticals.

### 4.2. Molecular Targets of Whole Pecan and Pecan Phenolic Extracts in Preventive and Intervention Strategies

The chronic consequences of the aforementioned alterations in metabolic tissues include NAFLD, type 2 diabetes and cardiovascular diseases (Figure 11A). Thus, an effective therapeutic approach for the prevention of chronic metabolic diseases must prevent the underlying causes through coordinated activities in several organs. In the present study, we demonstrated that consumption of whole pecans or a pecan polyphenol extract exerts beneficial effects in the different metabolic organs, consequently preventing the development of the chronic consequences of a high-fat diet.

Figure 11B shows the multifunctional targets of whole pecans or a pecan phenolic extract as observed in the present study. In this model we propose that whole pecans or pecan phenolic extracts exert their effects through a dual mode of action: a direct target effect and an indirect effect as a consequence of the former effect. Accordingly, we will discuss the benefits of pecans based on this dual mode of action. In the present study we identified five direct target effects and their corresponding indirect effects. In general, in the present study we showed that whole pecans and pecan phenolic extracts effectively prevented obesity (Figure 2A,B,E), NAFLD (Figure 8A,B), and type 2 diabetes (Figure 3B–F) when used as a preventive strategy.

One of the first targets of whole pecans or a pecan phenolic extract include a prebiotic effect, increasing bacterial diversity and preventing dysbiosis and endotoxemia, the latter considered an indirect effect (Figure 9). A second target is the up-regulation of PPARγ in both SAT and VAT and the increase in UCP-1 content in SAT (Figure 7). As a consequential indirect effect there was attenuation in fat accumulation, macrophage infiltration, lipolysis, reduced TNF alpha levels and an increase in adiponectin (Figure 7).

These first two targets of pecan bioactive had an indirect effect by attenuating the pro-inflammatory and lipotoxic milieu that in HF diet control mice is part of the early events taking place initiating as a low-grade inflammation state to convert later into a chronic inflammation state triggering the additional late metabolic alterations (Figure 11A,B).

A third target is the up-regulation of UCP-1 in brown adipose tissue that increases lipid oxidation and indirectly attenuates fat accumulation (Figure 5). A fourth target is the up-regulation of *p*-AMPK and *p*-AKT in muscle tissue (Figure 6F,G). Up-regulation of *p*-AMPK allowed mitochondria biogenesis that indirectly increased lipid oxidation while attenuating fat accumulation in muscle tissue (Figure 6A,B,C). On the other hand, up-regulation of *p*-AKT allowed the attenuation of insulin resistance (Figure 3A–F). In general, these third and fourth targets of pecan bioactive had as a consequential effect the increase in oxygen consumption and energy expenditure that contributed in the overall attenuation of fat accumulation in mice (Figure 2A,B,E,F and Figure 4). Furthermore, the fourth target of pecan bioactive in muscle tissue (through up-regulation of *p*-AKT) allowed an increase in glucose metabolism that favored the maintenance of lower serum glucose levels and indirectly attenuated islet hypertrophy in the pancreas and consequentially favored lower insulin levels in mice (Figure 2I and Figure 3A,G,H).

The fifth target is the up-regulation of *p*-AMPK and *p*-AKT in liver tissue that favored an increase in fatty acid oxidation attenuating steatosis and the development of NAFLD (Figure 8). Furthermore, as a consequence, the up-regulation of HMGCOAr gene expression favored lower levels of LDL in serum and indirectly reduced the risk of cardiovascular disease events (Figure 2G).

In the present study we identified targets 2 through 5 based on the criteria that up-regulation levels of the molecular markers were higher than those of the control samples and HF diet samples. For identifying target 1 the criteria were based on the maintenance of gut microbiome diversity as compared to HF diet controls.

In addition to the preventive study, we conducted an intervention study in obese mice for a period of 30 days using pecan phenolic extracts. In general, in the present study we showed that pecan phenolic extracts (6PP) partially reduced weight (Figure 10A,B,C), attenuated insulin resistance (Figure 10D,E,F), and increased energy expenditure (Figure 10G,H,I). Based on the direct target explanation described earlier, the observed weight loss plus the increase in lean tissue and reduced fat tissue would be due to pecan phenolic direct targets 2 (PPARγ and UCP-1), 3 (UCP-1) and 4 (*p*-AMPK and *p*-AKT), the effects involving visceral, subcutaneous and brown adipose tissue as well as muscle tissue. This reduced weight loss is in part due to the observed increase in energy expenditure involving brown adipose and muscle tissue and the corresponding pecan phenolic direct targets. On the other hand, the attenuation of insulin resistance would be associated to a direct target 4 effect involving muscle tissue that allowed a partial improvement of glucose metabolism.

According to the proposed model in Figure 11B, pecan phenolics in preventive or therapeutic strategies have the same targets and should be working in all tissues reverting the metabolic disorders; however, the intervention strategy only had a partial effect. One possible explanation is that the therapeutic partial effect could probably be due to the short intervention period applied. The idea is that, as long as the pro-inflammatory and lipotoxic milieu is present, all tissues are still challenged and the trigger is still on (Figure 11A). Thus, a key strategy would be to extend the intervention period until the pro-inflammatory and lipotoxic milieu is reduced to levels below low-grade inflammation and overcomes the feedback loop mechanism that sustains it. This implies that the extended intervention period would have to ensure that pecan phenolics have strong direct effects on targets 1 (prebiotic effect) and 2 (PPARγ and UCP-1). We propose that this extended intervention period will eventually increase further weight loss and reduce insulin resistance while reverting dysbiosis and the overall inflammation status. Further work is needed to test this hypothesis of pecan polyphenols in intervention strategies.

### 4.3. Whole Pecan and Pecan Phenolic Extracts and the Equivalent Dose for Future Clinical Studies

Pawnee pecans used in this study are a readily available source of polyunsaturated fatty acids and phenolic compounds [10,11]. The whole pecan treatment provided a similar phenolic concentration, 5.7 mg/g feed, to that of the 6PP pecan phenolic extract, 6 mg/g feed, while the 3PP extract provided 3.6 mg/g feed (Table 1). In general, the response of WP and 6PP was similar suggesting it is due mainly to the phenolic compounds present (Table 3); however, any slight differences could also be attributed to the pecan oil or fibers in WP. However, results with 3PP and 6PP do confirm that pecan phenolics are exerting the biological effects observed in this study due to the phenolic dose response observed.

To determine a reference dose for use in clinical settings, we used the body surface area (BSA) normalization method to convert the dose used in the present study in mice to a dose equivalent for human intake [49,50]. As indicated in the present study, consuming 3 g/day of the experimental diets containing whole pecans (WP) or pecan phenolic extracts (3PP, 6PP) provides mice with 17.1, 10.8 and 18 mg/day of bioactive phenolic compounds, respectively (Figure 2C, Table 1). In mice of ~25-g body weight, this amount of bioactive phenolics corresponds to 684, 432 and 720 mg phenolics/kg per day, which multiplied by the mouse Km factor (3) and divided by the human Km factor (37) gives a dose equivalent for humans of 55.4, 35 and 58.3 mg phenolics/kg per day, respectively. The Km factor is obtained by dividing body weight (kg) and BSA (m^2^) and is used for dose conversion of drugs between mg/kg and mg/m^2^, as described [49]. Thus, for an average 60 kg subject, the bioactive phenolic intake from whole pecans (WP) or pecan phenolic extracts (3PP, 6PP) would correspond to ~3327, 2101 and 3502 mg phenolics/day, respectively. Thus, considering, that “Pawnee” whole pecans contain ~1908.03 mg phenolic/100g pecans, then the calculated intake of whole pecans (WF) or pecan phenolic extracts (3PP, 6PP) would correspond to 174.3, 110.1 and 183.5g of pecan kernels, respectively and since “Pawnee” pecan kernels weight ~4.8 g (2 halves) [51], this amount of pecan kernels could be supplied by daily consumption of 36, 22 and 38 pecans, respectively. Alternatively, the daily pecan phenolic intake could also be supplied by consuming 34.2, 21.6 and 36 g of defatted pecan flour (considering an 80.37% fat content of pecan kernels), respectively. In general, the proposed intake of 110.1–183.5 g pecan kernels would provide 88.4–147.4 g of oil which is in a similar range to the Mediterranean diet fat intake that provides healthy fat up to 40% of total calories [52] for a typical daily calorie intake range of 2000–3000 kcal [53].

In summary, the present pre-clinical study has shown that to obtain more impactful results in clinical studies with pecans, the range of 42–90 g pecans used in previous studies would have to be increased to a range of 110.1–183.5 g pecans as shown in the present study. On the other hand, taking as reference the FDA daily recommendation of 42.5 g of nuts, our study suggests increasing the amount of whole pecan consumption by 2.5 to 4 times.

The mouse is a commonly used model for studying human physiology and metabolism, but it is important to note that conclusions derived from rodents may not necessarily translate directly to humans. However, pre-clinical studies are needed in order to establish the basis for clinical interventions and thus, translating basic findings to the clinic. On the other hand, one of the possible limitations for the consumption of pecan nuts is the presence of potential allergens such as Car i 1, Car i 4 and vicilin [54]. Certainly, food allergy can be a very serious concern in susceptible subjects. Therefore, it is mandatory to add precautionary advice on pecans and their derivatives. To reduce pecan food allergies, Clermont et al. [55] recently reported a proteomic methodology to assess allergenic protein content in different pecan cultivars, which may be useful for identifying low-allergenic pecan varieties. In general, it is unclear whether the same metabolic benefits would be observed in animals on a normal or low-fat diet, but further studies are warranted. Most importantly, through the duration and doses assayed, we did not find any negative effect of pecan or pecan polyphenols in any organ studied or the overall health of mice.

## 5. Conclusions

In the present study using mice fed a high-fat diet we demonstrated that whole pecans (*Carya illinoinensis*) or a pecan phenolic extract (mainly condensed tannin and ellagic acid derivatives) prevented obesity, liver steatosis and diabetes by reducing dysbiosis and inflammation, and increasing mitochondrial content and energy expenditure. Furthermore, the proposed model of the progression of the HF diet-mediated metabolic disorder based on early and late events was presented as a way to define the molecular target of the pecan treatments. Results indicated the molecular targets of whole pecans and pecan phenolic extracts based on preventive and intervention strategies on different organ tissues, confirming the multifunctionality of the pecan bioactive phenolics. Phenolics from pecans showed a pre-biotic effect preventing dysbiosis caused by HF and reducing pro-inflammatory serum LPS. This reduced anti-inflammatory activity was extended to visceral adipose tissue, preventing macrophage recruitment and TNF alpha synthesis and release. Furthermore, pecan polyphenols increased the activation of metabolic nuclear receptors such as PPAR gamma in adipose tissues and PPAR delta in skeletal muscle, in addition to the activation of *p*-AMPK. The activation of these master transcriptional regulators prevented adipocyte dysfunction and maintained or enhanced skeletal muscle mitochondrial activity despite a high-fat diet. Pecan phenolics also targeted up-regulation of UCP-1 in different adipose tissues that aid in an increase in energy expenditure. Furthermore, pecan phenolics also induced the activation of *p*-AKT which prevented glucose metabolism dysfunction and prevented pancreas dysfunction due to the attenuation of increased serum glucose levels. Up-regulation of *p*-AKT and *p*-AMPK in liver prevented steatosis and normalized cholesterol metabolism. In addition, pecan polyphenols were tested for a 30-day intervention in obese mice which showed a partial weight loss effect, a decrease in insulin resistance and an increase in energy expenditure. To have a more impactful effect we propose the need to reverse dysbiosis and the pro-inflammatory and lipotoxic milieu buildup that activated the early-stage response that in turn activated the late response.

Using the body surface area normalization method, the quantity of food consumed by the mice in the present study gave a conversion equivalent of 2101–3502 mg phenolic daily intake in humans. This amount can be obtained from 110–183 g pecan kernels/day (~22–38 whole pecans) or alternatively from 21.6–36 g defatted pecan flour/day for an adult of 60 kg. The results of the present study encourage the use of pecans as a non-pharmacological approach for the prevention or treatment of obesity and metabolic syndrome.

## Figures and Tables

**Table 1:** Components of the experimental diets.

Ingredients (g)	Control	HF	HF WP	HF 3PP	HF 6PP
Casein	20	20	18.2	20	20
Sucrose	10	31.6	29.8	30.6	30.0
Dextrose	13.2	15	15	15	15
Corn starch	39.75	0	0	0	0
Lard	0	16.4	0	16.4	16.4
Soy oil	7	7	0	7	7
Cellulose	5	5	2	5	5
Mineral mixture	1	1	1	1	1
Vitamin mixture	3.5	3.5	3.5	3.5	3.5
Cystine	0.3	0.3	0.3	0.3	0.3
Choline citrate	0.25	0.25	0.25	0.25	0.25
Whole pecan	0	0	30	0	0
Pecan phenolic extract	0	0	0	0.93	1.54

HF = High-fat diet; HF WP = High-fat diet Whole Pecan; HF PP = High-fat diet with Polyphenol Pecan. Phenolic content of whole pecan = 1908.03 mg phenolics/100 g kernels and pecan phenolic extract = 389 mg phenolics/g extract. Phenolic content of 3PP = 3.6 mg phenolics/g feed, 6PP = 6 mg phenolics/g feed and HF WP = 5.72 mg phenolics/g feed.

**Table 2:** Real time PCR forward and reverse primer sequences.

Gene	Forward Sequence (5′-3′)	Reverse Sequence (3′-5′)
SREBP-2	GATGATCACCCCGACGTTCA	GTCACGAGGCTTTGCACTTG
PPARδ	CTCTTCATCGCGGCCATCATTCT	TCTGCCATCTTCTGCAGCAGCTT
AMPK	ACCTGAGAACGTCCTGCTTG	GGCCTGCGTACAATCTTCCT
PPARγ2	CTCCTGTTGACCCAGAGCAT	GAAGTTGGTGGGCCAGAA
PGC-1a	AAGTGTGGAACTCTCTGGAACTG	GGGTTATCTTGGTTGGCTTTATG
HMGCOA	GGGTATTGCTGGCCTCTTCA	GGATTGCCATTCCACGAGCT

**Table 3:** LC-MS identification of phenolic compounds from Pawnee pecan (peaks from Figure 1).

Peak No	RT	M-H	* MS Fragments	Identification
1	14.87–14.96	577	451, 425, 407, **289**, 245	Procyanidin B2
1a	15.89–16.29	451	289	Catechin hexoside
2	17.00	433	301, 165	Ellagic acid pentoside
2a	17.82–17.89	477	315, **301**	Methyl ellagic acid hexoside
3	18.69–18.82	447	315, **301**	Methyl ellagic acid pentoside
4	19.49–19.56	615	463, **301**	Di-galloyl ellagic acid
4a	19.89	585	433, 301	Ellagic acid galloyl pentoside
5	20.36	599	447, **315**	Methyl ellagic acid galloyl pentose

* Note: Bold numbers indicate the base peaks in MSn spectra.

## Data Availability

Not applicable.

## Abbreviations

AMPK	Adenine monophosphate (AMP) activated protein kinase
BAT	Brown adipose tissue
BODIPY	4,4-difluoro-4-bora-3a,4a-diaza-s-indacene
CLA	Conjugated linoleic acid
DAPI	4′,6-Diamidino-2-Phenylindole, Dihydrochloride
H&E	Hematoxylin & Eosin
HF	High-fat
HMGCOA	5-hydroxy-3-methylglutaryl-coenzyme A) reductase
ipGTT	intraperitoneal Glucose Tolerance Test
ipITT	intraperitoneal Insulin Tolerance Test
mRNA	Messenger ribonucleic acid
PBS	Phosphate buffer saline
PCR	Polymerase chain reaction
PPARγ	Peroxisome proliferator-activated receptor gamma
PPARδ	Peroxisome proliferator-activated receptor delta
RER	Respiratory Exchange Ratio
SAT	Subcutaneous adipose tissue
SDH	Succinate Dehydrogenase
SREBP-2	Sterol regulatory element binding protein-2
TNF-α	Tumor necrosis factor alpha
UCP-1	Uncoupling protein one
VAT	Visceral adipose tissue
GLUT 4	Glucose Transporter 4
AKT	Serine/threonine-protein kinase
PGC-1 α	Peroxisome proliferator-activated receptor gamma coactivator 1 alpha
